# Supplementary material for: Characterization and Advancement of an Evaluation Method for the Treatment of Spontaneous Osteoarthritis in STR/ort Mice: GRGDS Peptides as a Potential Treatment for Osteoarthritis
Source: Biomedicines. 2023 Apr 6;11(4):1111. doi: 10.3390/biomedicines11041111 (PMC10136295; doi:10.3390/biomedicines11041111)
Supplement: Supplementary file 1 [file biomedicines-11-01111-s001.zip › biomedicines-2253034-supplementary.pdf]

Supplementary Information

Supplementary information accompanies this paper at additional files.

Additional files:

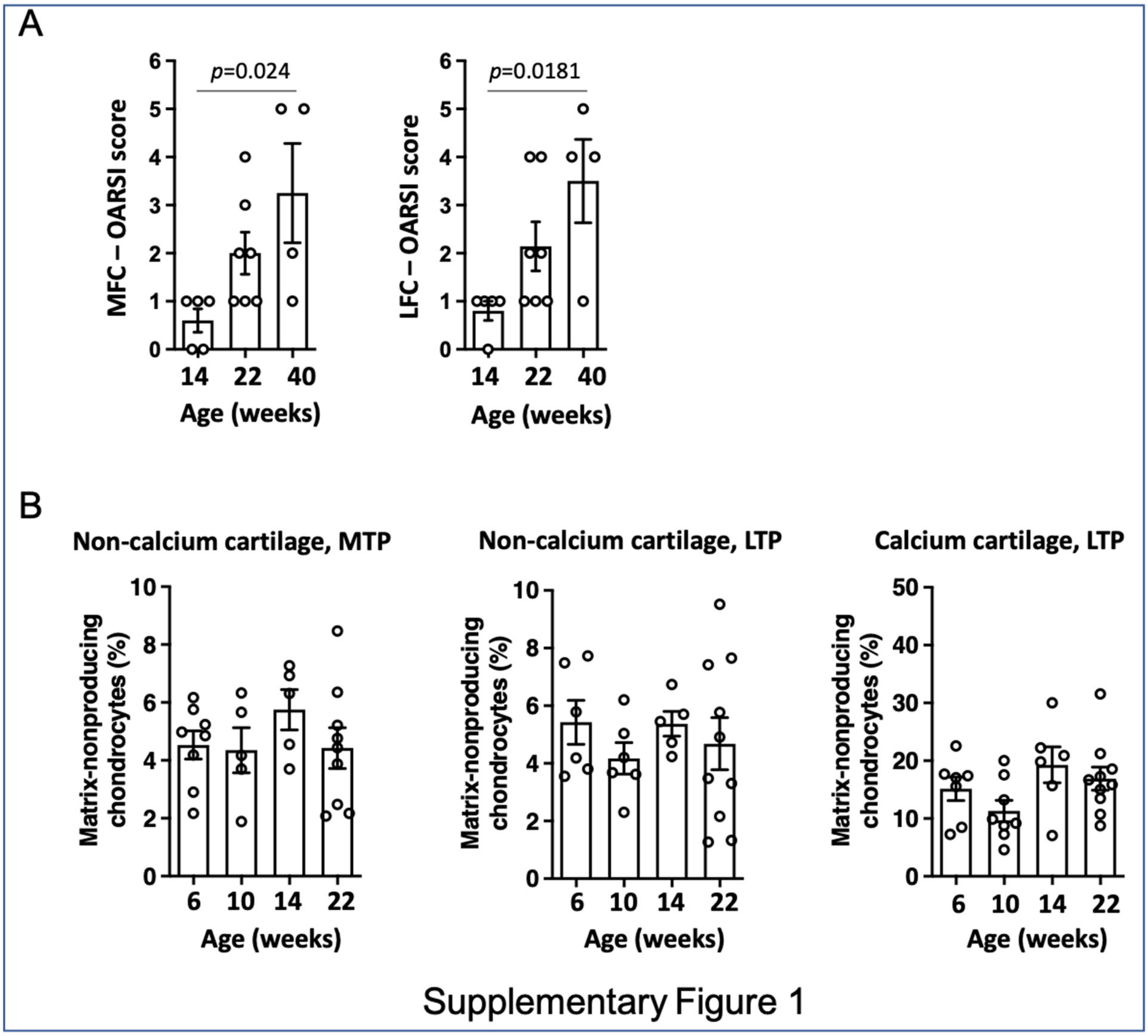

Supplementary Figure S1. The articular cartilage of male STR/ort mice showing varying OARSI score with age.

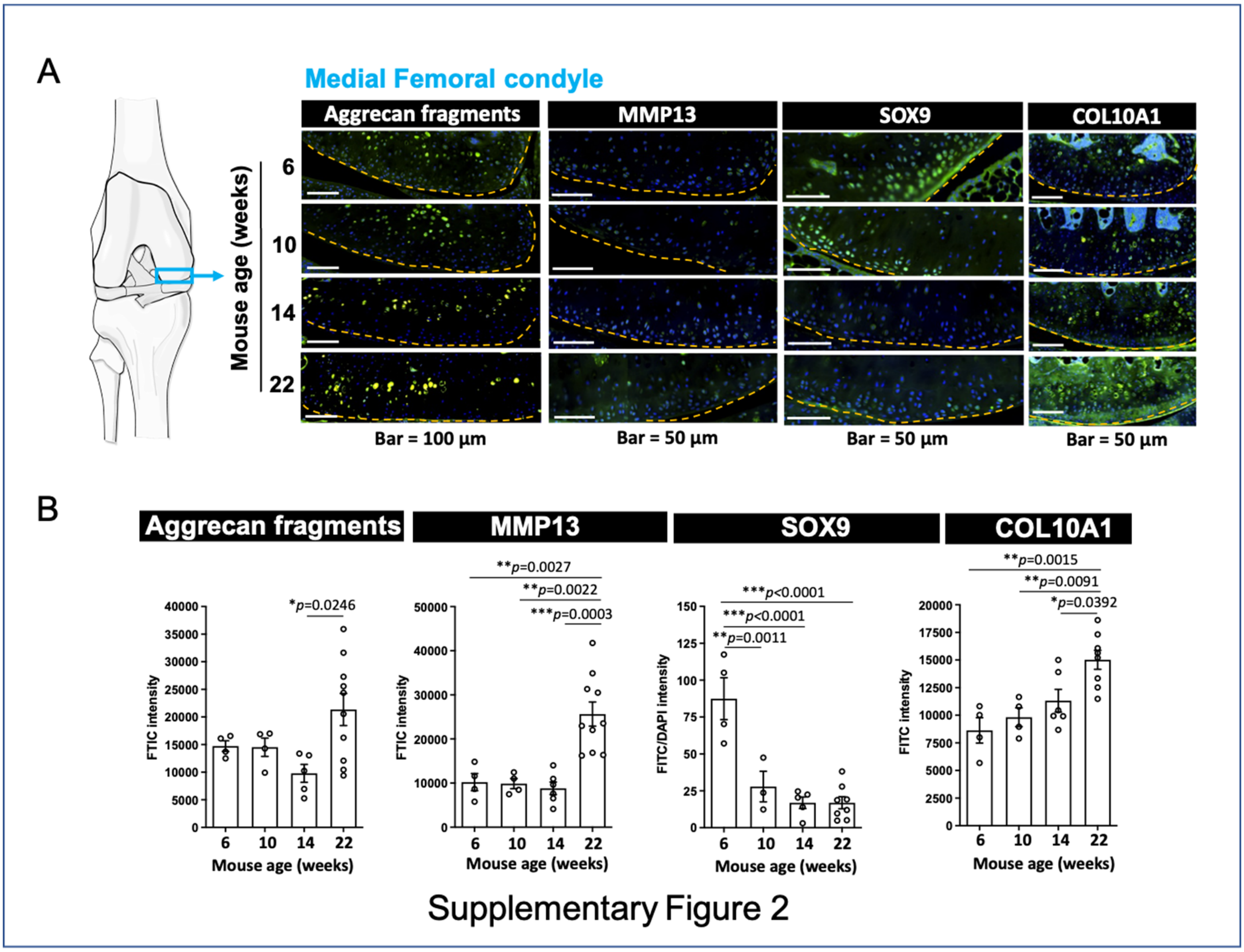

Supplementary Figure S2. Changed expression level of aggrecan fragments, MMP-13, Sox9, and COL10A1 expression associated with age is detected in OA cartilage.

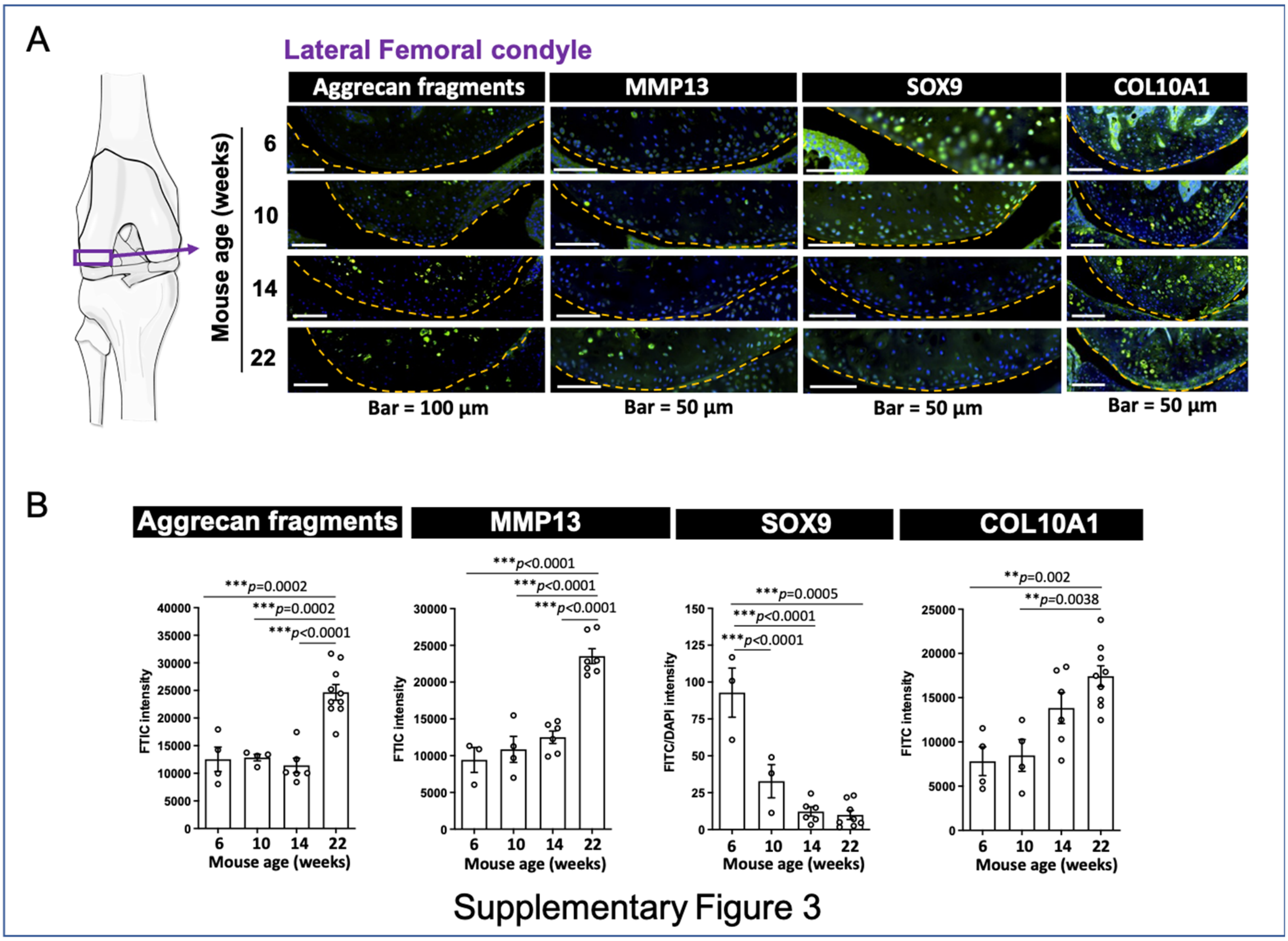

Supplementary Figure S3. Changed expression level of aggrecan fragments, MMP-13, Sox9, and COL10A1 expression associated with age is detected in OA cartilage.

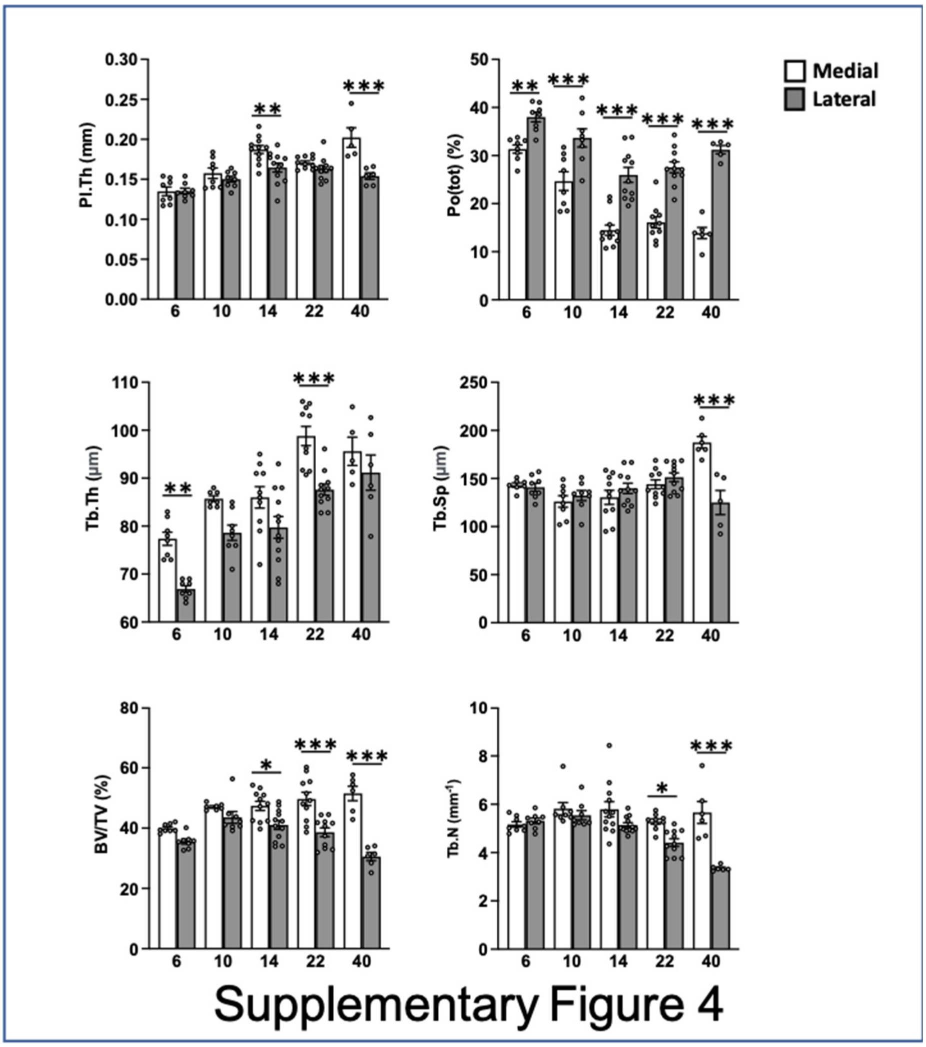

Supplementary Figure S4. Subchondral bone plate thickness and epiphyseal trabecular bone changed in the medial tibial plateau and lateral tibial plateau.

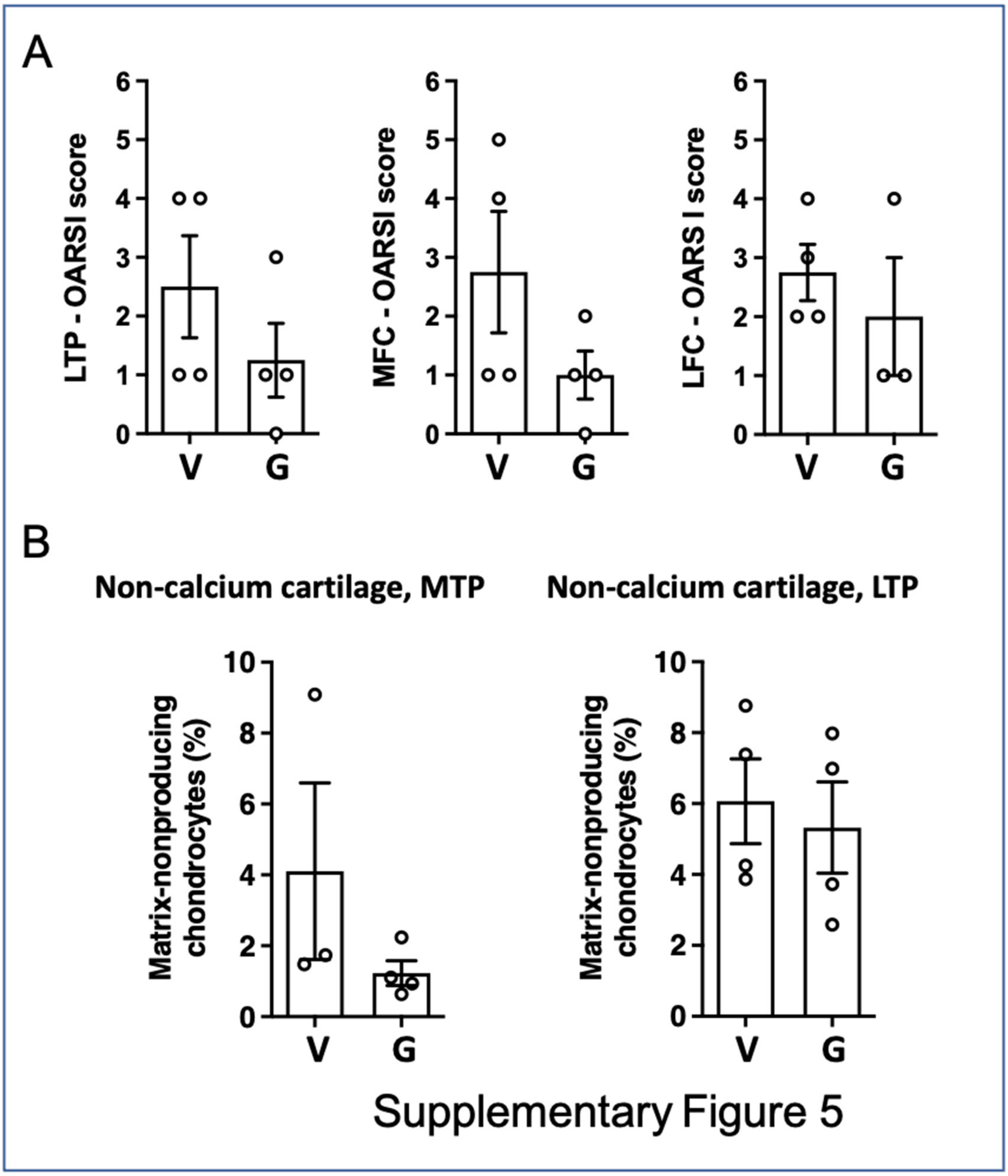

Supplementary Figure S5. GRGDS domain prevents OA progression in STR/ort mice.

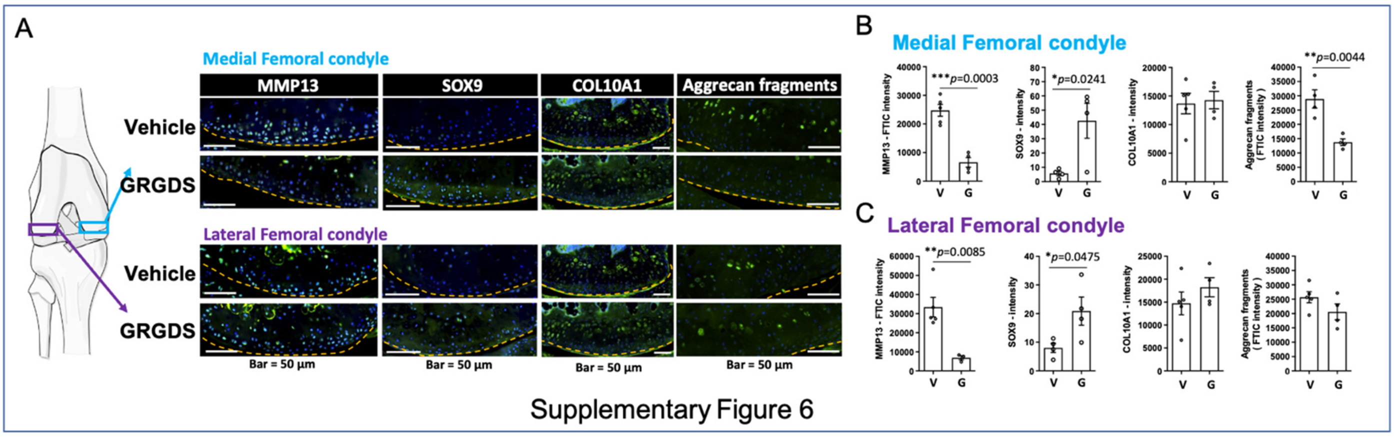

Supplementary Figure S6. GRGDS domain treatment attenuated processes associated with chondrocyte hypertrophy in the expression of MMP-13, Sox9, and aggrecan fragments.

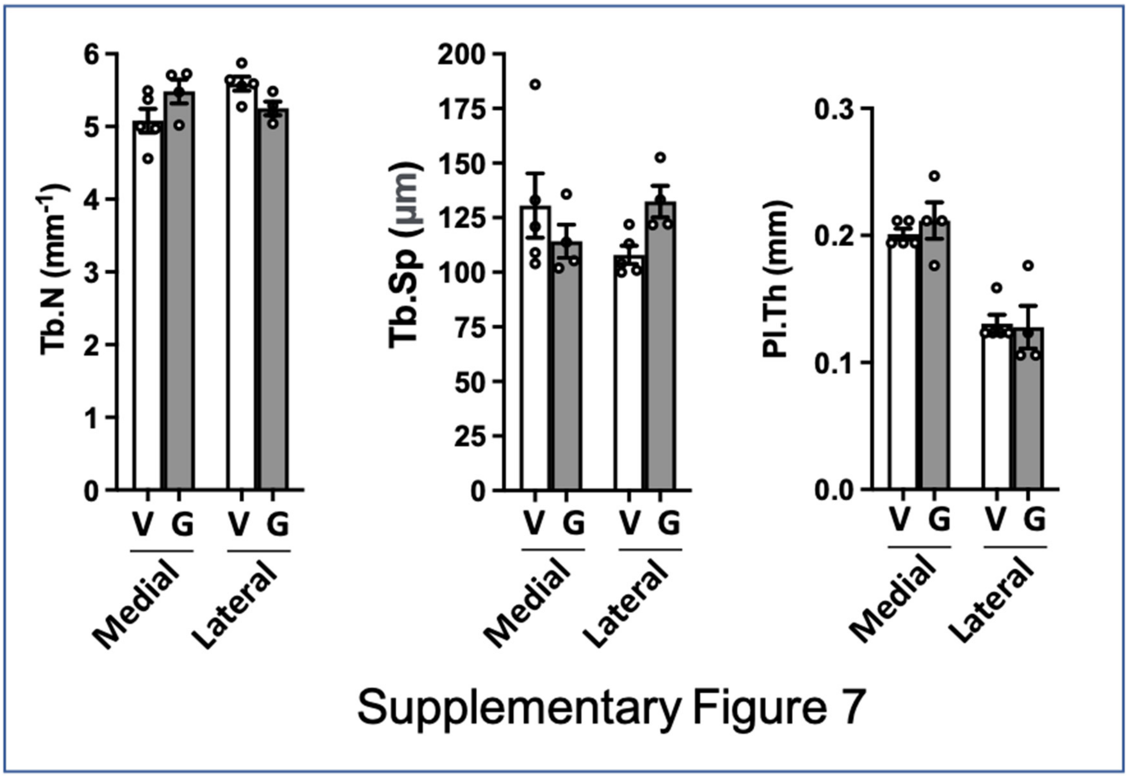

Supplementary Figure S7. Effects of GRGDS domain treatment on the bone remodeling in STR/ort knee joints.
